# Cost-Effectiveness of Lipid-Lowering Therapies for Cardiovascular Prevention in Germany

**DOI:** 10.1007/s10557-021-07310-y

**Published:** 2022-01-11

**Authors:** Daniel Tobias Michaeli, Julia Caroline Michaeli, Tobias Boch, Thomas Michaeli

**Affiliations:** 1 Fifth Department of Medicine, University Hospital Mannheim, Heidelberg University, Mannheim, Germany; 2 Department of Obstetrics and Gynecology, Asklepios-Clinic Hamburg Altona, Asklepios Hospital Group, Hamburg, Germany; 3 Department of Hematology and Oncology, University Hospital Mannheim, Heidelberg University, Mannheim, Germany; 4 Division of Personalized Medical Oncology, German Cancer Research Center (DKFZ), Heidelberg, Germany; 5 Department of Personalized Oncology, University Hospital Mannheim, Heidelberg University, Mannheim, Germany

**Keywords:** Cost-effectiveness, PCSK9 inhibitors, Icosapent ethyl, Ezetimibe, Statin

## Abstract

**Purpose:**

Novel pharmaceutical treatments reducing cardiovascular events in dyslipidaemia patients must demonstrate clinical efficacy and cost-effectiveness to promote long-term adoption by patients, physicians, and insurers.

**Objective:**

To assess the cost-effectiveness of statin monotherapy compared to additive lipid-lowering therapies for primary and secondary cardiovascular prevention from the perspective of Germany’s healthcare system.

**Methods:**

Transition probabilities and hazard ratios were derived from cardiovascular outcome trials for statin combinations with icosapent ethyl (REDUCE-IT), evolocumab (FOURIER), alirocumab (ODYSSEY), ezetimibe (IMPROVE-IT), and fibrate (ACCORD). Costs and utilities were retrieved from previous literature. The incidence of major adverse cardiovascular events was simulated with a Markov cohort model. The main outcomes were the incremental cost-effectiveness ratios (ICER) per quality adjusted life year (QALY) gained.

**Results:**

For primary prevention, the addition of icosapent ethyl to statin generated 0.81 QALY and €14,732 costs (ICER: 18,133), whereas fibrates yielded 0.63 QALY and € − 10,516 costs (ICER: − 16,632). For secondary prevention, the addition of ezetimibe to statin provided 0.61 QALY at savings of € − 5,796 (ICER: − 9,555) and icosapent ethyl yielded 0.99 QALY and €14,333 costs (ICER: 14,485). PCSK9 inhibitors offered 0.55 and 0.87 QALY at costs of €62,722 and €87,002 for evolocumab (ICER: 114,639) and alirocumab (ICER: 100,532), respectively. A 95% probability of cost-effectiveness was surpassed at €20,000 for icosapent ethyl (primary and secondary prevention), €119,000 for alirocumab, and €149,000 for evolocumab.

**Conclusions:**

For primary cardiovascular prevention, a combination therapy of icosapent ethyl plus statin is a cost-effective use of resources compared to statin monotherapy. For secondary prevention, icosapent ethyl, ezetimibe, evolocumab, and alirocumab increase patient benefit at different economic costs.

**Supplementary Information:**

The online version contains supplementary material available at 10.1007/s10557-021-07310-y.

## Introduction

Cardiovascular diseases (CVD) remain the leading cause of death in Germany, accounting for more than 35% of all fatalities [1]. Besides adverse health effects for the individual, CVD lead to rising medical costs for society [2]. The American Heart Association identified the ongoing demographic shift, increasing prevalence, and rising treatment costs as main drivers for surging CVD expenses [3].

For patients with elevated triglycerides and cholesterol, treatment strategies for primary and secondary cardiovascular prevention include lipid-lowering drugs. Since the launch of statins more than 25 years ago, several additive therapies were introduced. However, only ezetimibe, icosapent ethyl, evolocumab, and alirocumab significantly reduced the risk of major adverse cardiovascular events (MACE) by 6% (95% CI: 1 to 11, *p* = 0.016), 25% (95% CI: 17 to 32, *p* < 0.001), 15% (95% CI: 8 to 21, *p* < 0.001), and 15% (95% CI: 7 to 22, *p* < 0.001), respectively [4–7].

In line with each drug’s label, the European Society of Cardiology (ESC) recommends individualized dyslipidaemia treatment strategies depending on patient-specific plasma cholesterol and triglyceride levels. This distinction matters as elevated low-density lipoprotein cholesterol (LDL-C) levels adversely impact 10-year patient survival. For the purposes of our analyses, we therefore categorized treatment options in cholesterol lowering (ezetimibe, alirocumab, and evolocumab) and triglyceride lowering (icosapent ethyl and fibrate) strategies based on ESC guidelines [8].

### Cholesterol Lowering Strategy

A combination of statin plus ezetimibe is indicated for very high-risk patients who do not achieve their cholesterol treatment goals under the maximum tolerable statin monotherapy. If the predefined LDL-C levels are still not attained under an ezetimibe plus statin combination, doctors may further prescribe proprotein convertase subtilisin-kexin type 9 (PCSK9) inhibitors.

### Triglyceride Lowering Strategy

In contrast, icosapent ethyl plus statin is recommended in high-risk patients with elevated triglyceride levels despite statin monotherapy treatment. Similarly, fibrates may be used to supplement statin monotherapy in high-risk patients with elevated triglyceride levels.

The recent launch of icosapent ethyl in Europe combined with pricing disputes surrounding PCSK9 antibodies and genericization of ezetimibe warrant a cost-effectiveness analysis of existing lipid-lowering therapies. This study examines the cost-effectiveness of additive lipid-lowering therapies compared to statin monotherapy for primary and secondary cardiovascular prevention from the perspective of the German healthcare system. Whilst previous studies mostly focused on one particular drug for secondary prevention, this is the first study assessing the clinical economics of five lipid-lowering therapeutics in both primary and secondary prevention.

## Data and Methods

### Model Structure

**Fig. 1:**
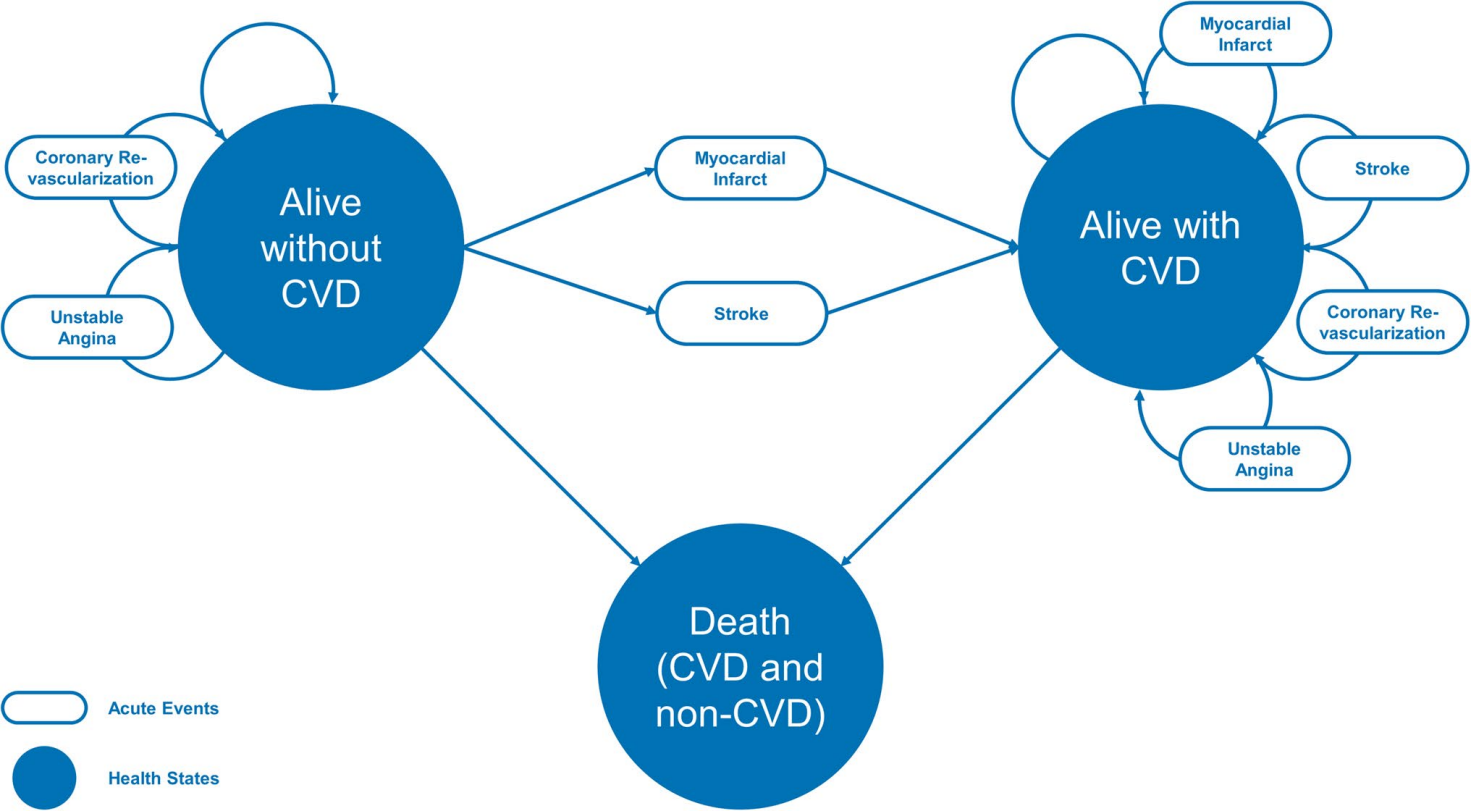
Markov model structure of health states and acute cardiovascular events. The Markov model illustrates health states of the stimulated cohort defined as “Alive without CVD”, “Alive with CVD”, and “Death”. In any cycle, individuals were at risk for acute cardiovascular events: myocardial infarct, stroke, coronary revascularization, and unstable angina. *CVD* cardiovascular disease.

A Markov model comparing statin monotherapy with statin combination therapies (icosapent ethyl, PCSK9 inhibitors, ezetimibe, and fibrate) for cardiovascular prevention was constructed with Microsoft Excel (2016). The model entails three underlying health states: “Alive without CVD”, “Alive with CVD”, and “Dead” (Fig. 1). Patients without CVD were at risk to experience a non-fatal myocardial infarct (MI) or non-fatal stroke, which directed them to the “Alive with CVD” state. All alive patients were also at risk to dying from CVD or non-CVD causes, which channelled them to the death state. All alive patients were at risk for hospitalization for unstable angina and coronary revascularization, yet these events did not alter their underlying health state. Primary prevention patients started the model in the “Alive without CVD” state, whereas secondary prevention patients began the model in the “Alive with CVD state”. In line with the Institute for Quality and Efficiency in Health Care (IQWiG) guidelines, the model was constructed from the perspective of the German healthcare system with a time horizon of 20 years and a discount rate of 3% for utilities and costs [9].

### Evaluated Treatment Options

All lipid-lowering drugs that were approved by the European Medicines Agency (EMA) for primary or secondary cardiovascular prevention in combination treatment with statins after the year 2000 were included. For every metabolic agent, the largest (in terms of enrolled patients) available randomized clinical trial reporting MACE was considered. Icosapent ethyl and fibrate were the only treatments with available data for primary cardiovascular prevention. Ezetimibe, icosapent ethyl, evolocumab, alirocumab, and fibrate were evaluated for secondary cardiovascular prevention. No endpoint studies were completed for bile acid sequestrants, inclisiran, and bempedoic acid to date. A fixed combination of nicotinic acid and laropiprant was excluded due to the negative recommendation issued by the EMA in 2012 following the HPS2-THRIVE study [10].

### Transition Probabilities

Transition probabilities were derived from cardiovascular outcome trials for icosapent ethyl (REDUCE-IT), evolocumab (FOURIER), alirocumab (ODYSSEY), ezetimibe (IMPROVE-IT), and fibrate (ACCORD) [4–7, 11]. All trials reported separate outcomes for non-fatal MI, non-fatal stroke, hospitalization for unstable angina, coronary revascularization, CVD death, and non-CVD death. Extracted outcomes were transformed to 1-year transition probabilities based on the median follow-up time from cardiovascular outcome trials (Table 1).

**Table 1:** Model input parameters: base case value, variation, and distribution for hazard ratios, utilities, and costs

Parameter	Icosapent Ethyl			Evolocumab			Alirocumab			Ezetimibe				Distribution
	Value	Variation	Ref	Value	Variation	Ref	Value	Variation	Ref	Value	Variation	Ref		
**Hazard ratios**														
Alive without CVD (primary prevention)														
Non-fatal MI	0.78	(0.67, 0.90)	[5]	NA	NA	[6]	NA	NA	[7]	NA	NA	[4]		Normal
CVD death	0.90	(0.77, 1.04)	[5]	NA	NA	[6]	NA	NA	[7]	NA	NA	[4]		Normal
Non-CVD death	1.14	(0.97, 1.31)	[5]	NA	NA	[6]	NA	NA	[7]	NA	NA	[4]		Normal
Non-fatal stroke	0.81	(0.69, 0.93)	[5]	NA	NA	[6]	NA	NA	[7]	NA	NA	[4]		Normal
Hospitalization for unstable angina	0.76	(0.64, 0.87)	[5]	NA	NA	[6]	NA	NA	[7]	NA	NA	[4]		Normal
Coronary revascularization	0.76	(0.64, 0.87)	[5]	NA	NA	[6]	NA	NA	[7]	NA	NA	[4]		Normal
Alive with CVD (secondary prevention)														
Non-fatal MI	0.68	(0.58, 0.79)	[5]	0.72	(0.64, 0.81)	[6]	0.86	(0.77, 0.96)	[7]	0.87	(0.80, 0.95)	[4]		Normal
CVD death	0.79	(0.67, 0.91)	[5]	1.05	(0.88, 1.25)	[6]	0.88	(0.74, 1.05)	[7]	1.00	(0.89, 1.13)	[4]		Normal
Non-CVD death	1.01	(0.85, 1.16)	[5]	1.04	(0.91, 1.19)	[6]	0.78	(0.66, 0.94)	[7]	0.98	(0.90, 1.06)	[4]		Normal
Non-fatal stroke	0.71	(0.60, 0.82)	[5]	0.91	(0.78, 1.07)	[6]	0.73	(0.57, 0.93)	[7]	0.86	(0.73, 1.00)	[4]		Normal
Hospitalization for unstable angina	0.67	(0.57, 0.77)	[5]	0.99	(0.82, 1.18)	[6]	0.61	(0.41, 0.92)	[7]	1.06	(0.85, 1.33)	[4]		Normal
Coronary revascularization	0.66	(0.56, 0.75)	[5]	0.78	(0.71, 0.86)	[6]	0.88	(0.79, 0.97)	[7]	0.96	(0.90, 1.02)	[4]		Normal
**Utilities**														
Alive without CVD														
65–70 years	0.65	(0.64, 0.67)	[13]	0.65	(0.64, 0.67)	[13]	0.65	(0.64, 0.67)	[13]		0.65	(0.64, 0.67)	[13]	Beta
70 + years	0.63	(0.62, 0.65)	[13]	0.63	(0.62, 0.65)	[13]	0.63	(0.62, 0.65)	[13]	0.63	(0.62, 0.65)	[13]		Beta
Alive with CVD														
65–70 years	0.57	(0.56, 0.59)	[14]	0.57	(0.56, 0.59)	[14]	0.57	(0.56, 0.59)	[14]	0.57	(0.56, 0.59)	[14]		Beta
70 + years	0.55	(0.54, 0.57)	[14]	0.55	(0.54, 0.57)	[14]	0.55	(0.54, 0.57)	[14]	0.55	(0.54, 0.57)	[14]		Beta
Decrements														
Non-fatal MI	0.04	(0.02, 0.05)	[44]	0.04	(0.02, 0.05)	[44]	0.04	(0.02, 0.05)	[44]	0.04	(0.02, 0.05)	[44]		Gamma
Non-fatal stroke	0.12	(0.09, 0.16)	[44]	0.12	(0.09, 0.16)	[44]	0.12	(0.09, 0.16)	[44]	0.12	(0.09, 0.16)	[44]		Gamma
Hospitalization for unstable angina	0.09	(0.06, 0.13)	[44]	0.09	(0.06, 0.13)	[44]	0.09	(0.06, 0.13)	[44]	0.09	(0.06, 0.13)	[44]		Gamma
Coronary revascularization	0.01	(0.01, 0.03)	[44]	0.01	(0.01, 0.03)	[44]	0.01	(0.01, 0.03)	[44]	0.01	(0.01, 0.03)	[44]		Gamma
**Costs**														
Alive without CVD	4,522	(± 25%)	[15]	4,522	(± 25%)	[15]	4,522	(± 25%)	[15]	4,522	(± 25%)	[15]		Gamma
Alive with CVD	8,008	(± 25%)	[16, 17]	8,008	(± 25%)	[16, 17]	8,008	(± 25%)	[16, 17]	8,008	(± 25%)	[16, 17]		Gamma
Non-fatal MI	8,588	(± 25%)	[17]	8,588	(± 25%)	[17]	8,588	(± 25%)	[17]	8,588	(± 25%)	[17]		Gamma
CVD death	11,842	(± 25%)	[16–18, 20]	11,842	(± 25%)	[16–18, 20]	11,842	(± 25%)	[16–18, 20]	11,842	(± 25%)	[16–18, 20]		Gamma
Non-CVD death	3,924	(± 25%)	[18–20]	3,924	(± 25%)	[18–20]	3,924	(± 25%)	[18–20]	3,924	(± 25%)	[18–20]		Gamma
Non-fatal stroke	10,441	(± 25%)	[17]	10,441	(± 25%)	[17]	10,441	(± 25%)	[17]	10,441	(± 25%)	[17]		Gamma
Hospitalization for unstable angina	4,791	(± 25%)	[16]	4,791	(± 25%)	[16]	4,791	(± 25%)	[16]	4,791	(± 25%)	[16]		Gamma
Coronary revascularization	9,901	(± 25%)	[16]	9,901	(± 25%)	[16]	9,901	(± 25%)	[16]	9,901	(± 25%)	[16]		Gamma
Annual treatment cost	2,400		[22]	5,880		[21]	7,509		[21]	156		[21]		Fixed
Annual statin cost	132		[21]	132		[21]	132		[21]	132		[21]		Fixed
**Others**														
Discount rate	0.03	(0.00, 0.10)	[9]	0.03	(0.00,0.10)	[9]	0.03	(0.00,0.10)	[9]	0.03	(0.00,0.10)	[9]		Fixed
Annual CVD risk increase	0.14	(± 25%)	[45]	0.14	(± 25%)	[45]	0.14	(± 25%)	[45]	0.14	(± 25%)	[45]		Fixed
Annual non-CVD risk increase	0.10	(± 25%)	[45]	0.10	(± 25%)	[45]	0.10	(± 25%)	[45]	0.10	(± 25%)	[45]		Fixed

The table displays each drug’s key input parameters used for the base case scenario of the cost-effectiveness model. Variation columns display upper and lower bound of parameter values that were used for sensitivity analyses. For hazard ratios and utilities, this variation equates to 95% confidence interval extracted from cited references. All costs are presented in Euros (€) and are inflation adjusted to 2021 values. Transition probabilities for the model are presented in Supplement Table 1. Data for fibrates are included in Supplement Table 2. No clinical trial data was available for ezetimibe, alirocumab, and evolocumab in the primary prevention setting. *CVD* cardiovascular disease, *MI* myocardial infarct, *NA* not applicable.

Evolocumab, alirocumab, and ezetimibe therapies only investigated cardiovascular protection in patient populations with diagnosed CVD. Consequently, transition probabilities were only applied to the secondary prevention model. The REDUCE-IT and ACCORD trial analyzed cardiovascular risk protection of icosapent ethyl and fibrate in both primary and secondary prevention. Therefore, separate transition probabilities for primary and secondary prevention were calculated based on a methodology described by Ademi et al. (2020) [12]. The methodology derived distinct probabilities based on the baseline prevalence of CVD, primary composite outcomes for CVD and non-CVD patients, and relative risk of events for CVD and non-CVD patients.

One-year transition probabilities were employed for the first cycle period. Thereafter, probabilities adjust for the increased CVD and non-CVD risks derived from German mortality data. Cardiovascular and non-cardiovascular risks were increased by 14% and 10% per year, respectively.

### Model Population

The model population features distinct patient populations and transition probabilities for each evaluated treatment alternative. Coherent with the weighted average patient age of all examined trials, the simulated cohort entered the model at age 63.

### Utilities

Age-specific health-related quality of life values for the “Alive without CVD” state were extracted from a longitudinal survey of 7,708 individuals in Germany [13]. The utility value of the “Alive with CVD” state was subsequently reduced by − 0.08 [14]. We assigned a utility value of 0 to the “Death” state.

Upon the occurrence of adverse cardiovascular events, the health-related quality of life was reduced for the respective cycle. Utility decrements were applied for non-fatal MI (− 0.04), non-fatal stroke (− 0.12), hospitalization for angina (− 0.09), and coronary revascularization (− 0.01) [12].

### Costs

The economic burden of cardiovascular disease and adverse events in Germany was extracted from previous literature. Annual costs for the “Alive without CVD” state of €4,522 are based on the cost of treating hypertriglyceridemia and related comorbidities, e.g. diabetes and hypertension [15]. Treatment costs for non-fatal cardiovascular events were extracted from peer-reviewed publications [16, 17]. The annual treatment costs of the “Alive with CVD” were estimated at €8,088. This estimate is based on the average treatment cost of CVD 1-year post occurrence [16]. Consequently, the cost of MI, strokes, heart failure, and peripheral artery disease were weighted by their respective prevalence among the secondary prevention cohort in the REDUCE-IT, IMPROVE-IT, FOURIER, and ODYSSEY trials.

We estimated costs associated with dying based on the location and cause of death. Expenses were weighted by the location of death as a German large-scale study (*n* = 59,922) found that 71% of patients die in hospitals/institutions and 29% at home [18]. Non-CVD hospital deaths were approximated with the average expense of hospital stays in Germany (€4,968) [19], while CVD hospital deaths are based on the weighted expense of fatal MI, strokes, and ischaemic heart disease (€16,120) [17]. Expenses for the last week of life at home amount to €1,369 in Germany [20]. All costs were adjusted for inflation to 2021 values.

Annual treatment costs for statins (€131.62), ezetimibe (€156.48), evolocumab (€5,879.54), alirocumab (€7,508.88), and fibrate (€130.44) were extracted from a German statutory insurance price schedule [21]. Annual treatment costs for icosapent ethyl (€2,400) were set based on manufacturer guidance [22].

### Outcomes

The principal outcomes were the incremental cost-effectiveness ratios (ICER) derived from quality-adjusted life years (QALY) and life years (LY). Numbers needed to treat (NNT) were furthermore compared across events and treatment strategies.

### Scenario, Sensitivity, Willingness-to-Pay, and Pricing Analyses

First, uncertainty surrounding input parameters was assessed in a univariate (one-way) sensitivity analysis. In addition, the impact of variations in drug price, discount rate, time horizon, and mortality trends was examined in a scenario analysis. A probabilistic sensitivity analysis (PSA) featuring 1,000 runs evaluates uncertainty of input parameters concurrently. A willingness-to-pay (WTP) analysis was conducted to estimate the ICER at which the probability of treatments being cost-effective surpasses 95%. Lastly, we evaluated the impact of drug pricing on calculated cost-effectiveness ratios (Fig. 1).

## Results

### Base Case Analysis

**Fig. 2:**
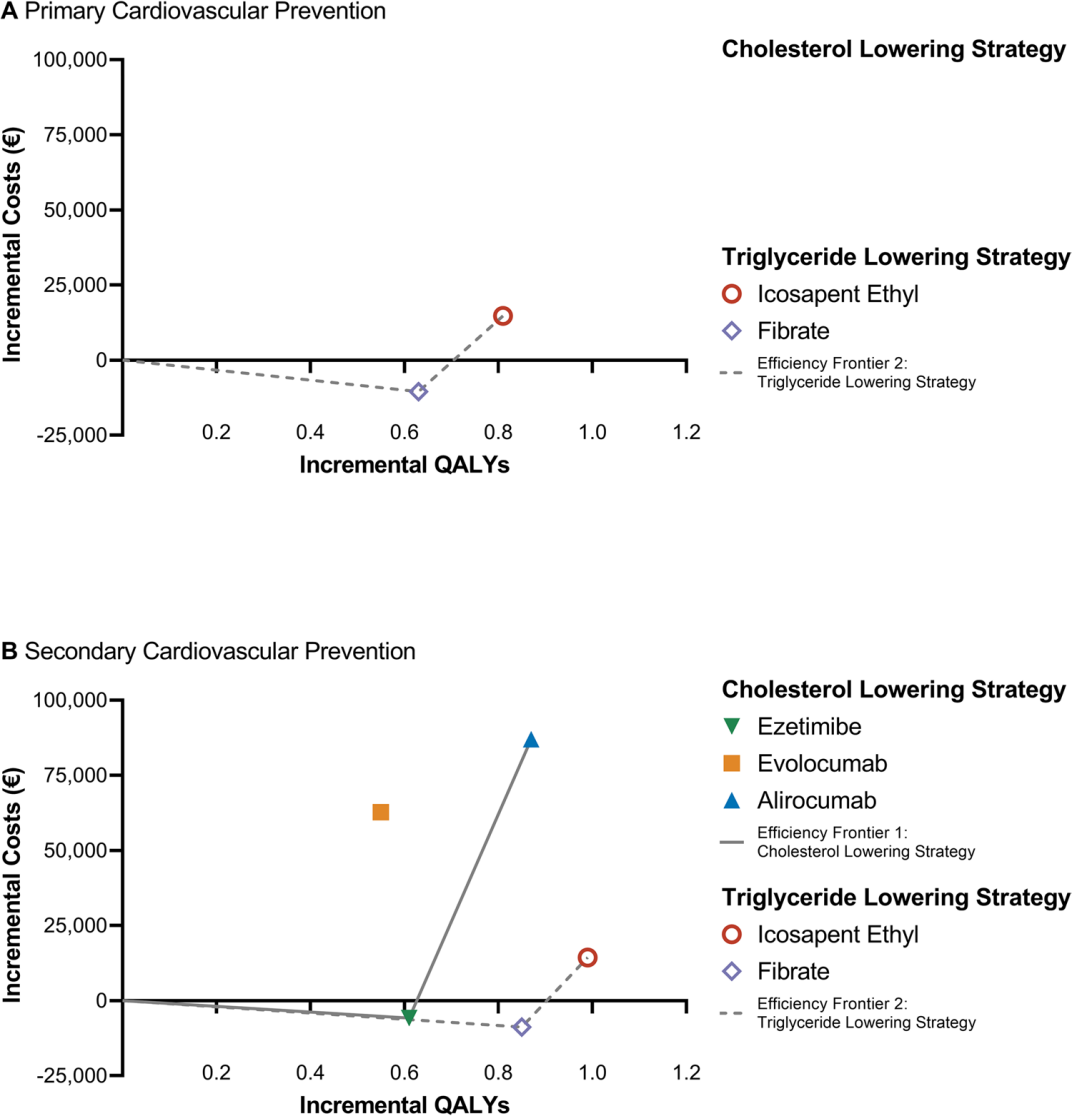
Cost-effectiveness plane with efficiency frontier for cholesterol and triglyceride lowering treatment strategies in combination with statin. Results are visualized for primary (**A**) and secondary cardiovascular prevention (**B**). All costs are presented in Euros (€) and inflation adjusted to 2021 values. QALYs and costs are displayed per person. Treatment options were categorized in cholesterol lowering (ezetimibe, alirocumab, evolocumab) and triglyceride lowering (icosapent ethyl, fibrate) strategies based on the European Society of Cardiology guidelines [8]. *QALY* quality-adjusted life year

Base case results are presented on the cost-effectiveness plane in Fig. 2. In the primary prevention setting, the addition of icosapent ethyl to statin generated 0.81 additional QALY and €14,732 costs (ICER: 18,133 €/QALY). The addition of fibrate to statin yielded 0.63 incremental QALY and € − 10,516 costs (ICER: − 16,632 €/QALY). The NNT for non-fatal MI (1.6 vs. 3.3), non-fatal strokes (5.6 vs. 16.3), and CVD deaths (3.9 vs. 5.0) were lower for icosapent ethyl compared to fibrates (Table 2).

**Table 2:** Model base case results over a 20-year time horizon for primary and secondary cardiovascular prevention

	Cholesterol lowering strategy			Triglyceride lowering strategy	
	Ezetimibe	Evolocumab	Alirocumab	Icosapent ethyl	Fibrate
**Primary prevention**					
Incremental QALYs	NA	NA	NA	0.81	0.63
Incremental LYs	NA	NA	NA	0.97	0.91
Incremental costs	NA	NA	NA	14,732	− 10,516
ICER (costs/LY)	NA	NA	NA	15,130	− 11,605
ICER (costs/QALY)	NA	NA	NA	18,133	− 16,632
Number needed to treat (NNT)					
Non-fatal MI	NA	NA	NA	1.6	3.3
Non-fatal stroke	NA	NA	NA	5.6	16.3
Hospitalization for unstable angina	NA	NA	NA	4.6	4.4
Coronary revascularization	NA	NA	NA	1.3	1.2
CVD death	NA	NA	NA	3.9	5.0
Non-CVD death	NA	NA	NA	41.7	17.5
**Secondary prevention**					
Incremental QALYs	0.61	0.55	0.87	0.99	0.85
Incremental LYs	0.86	0.65	1.23	1.34	1.37
Incremental costs	− 5,796	62,722	87,002	14,333	− 8,787
ICER (costs/LY)	− 6,711	96,243	71,005	10,695	− 6,427
ICER (costs/QALY)	− 9,555	114,639	100,532	14,485	− 10,305
Number needed to treat (NNT)					
Non-fatal MI	2.7	1.6	1.6	1.5	3.5
Non-fatal stroke	8.8	6.4	6.4	5.3	21.5
Hospitalization for unstable angina	31.3	5.8	14.1	4.3	5.2
Coronary revascularization	1.7	1.1	1.4	1.2	1.3
CVD death	7.3	6.4	4.5	3.8	5.2
Non-CVD death	15.8	24.3	17.9	48.8	23.1

All costs are presented in Euros (€) and inflation adjusted to 2021 values. QALYs, LYs, and ICERs are displayed per person. No clinical trial data was available for ezetimibe, alirocumab, and evolocumab in the primary prevention setting. Treatment options were categorized in cholesterol lowering (ezetimibe, alirocumab, evolocumab) and triglyceride lowering (icosapent ethyl, fibrate) strategies based on the European Society of Cardiology guidelines [8]. *QALY* quality-adjusted life year, *LY* life year, *ICER* incremental cost-effectiveness ratio, *NNT* number needed to treat, *CVD* cardiovascular disease, *MI* myocardial infarct, *NA* not applicable.

In the secondary prevention setting, the addition of ezetimibe to statin generated 0.61 incremental QALY at savings of € − 5,796 (ICER: − 9,555 €/QALY). PCSK9 inhibitors offered QALY gains of 0.55 and 0.87 at costs of €62,722 and €87,002 for evolocumab (ICER: 114,639 €/QALY) and alirocumab (ICER: 100,532 €/QALY), respectively. Icosapent ethyl provided a QALY gain of 0.99 at an expense of €14,333 (ICER: 14,485 €/QALY). The NNT for non-fatal MI (1.5 vs. 1.6/1.6), non-fatal strokes (5.3 vs. 6.4/6.4), and CVD deaths (3.8 vs. 6.4/5.4) were lower for icosapent ethyl compared to evolocumab/alirocumab.

### Sensitivity Analyses

Results of the univariate sensitivity analysis are presented in Supplement Figues e2 an e3. This analysis assesses the impact of one-way variations in a single input parameter on the drugs’ ICER. ICER were mainly impacted by variations in the cost of and transition probability to CVD death alongside the cost of the “Alive with CVD” state.

**Fig. 3:**
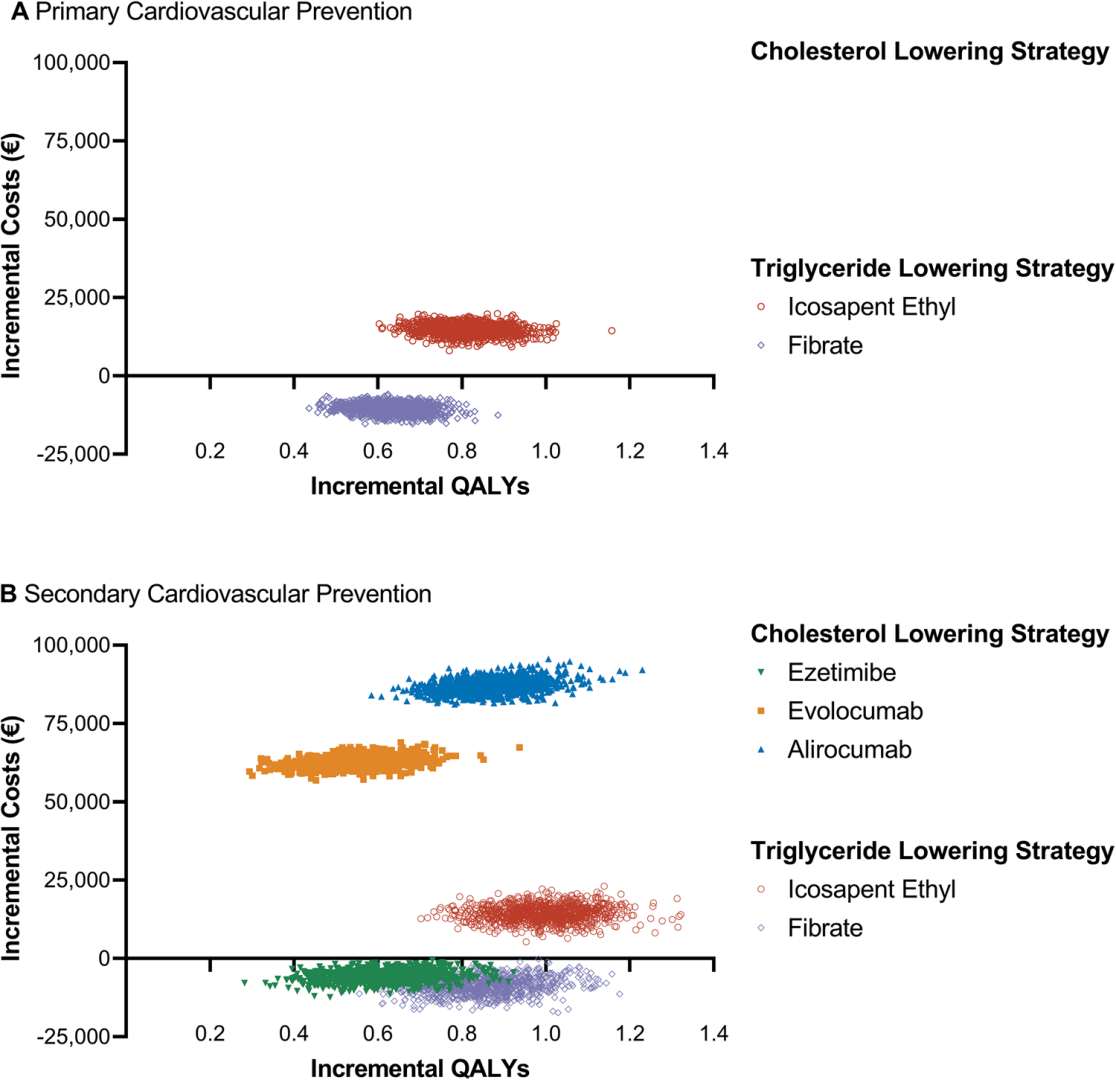
Probabilistic sensitivity analysis for cholesterol and triglyceride lowering treatment strategies in combination with statin displayed on a cost-effectiveness plane. Results are visualized for primary (**A**) and secondary cardiovascular prevention (**B**). All costs are presented in Euros (€) and were inflation adjusted to 2021 values. The figure displays 1,000 iterations of the conducted probabilistic sensitivity analysis which simultaneously varies input parameters by their confidence intervals and defined distribution presented in Table 1. Treatment options were categorized in cholesterol lowering (ezetimibe, alirocumab, evolocumab) and triglyceride lowering (icosapent ethyl, fibrate) strategies based on the European Society of Cardiology guidelines [8]. QALYs and costs are displayed per person. *QALY* quality-adjusted life year

The PSA with 1,000 iterations is displayed in Fig. 3. The simulation yielded mean ICER of 18,330 €/QALY (95% CI: 13,643 to 23,374) for icosapent ethyl and -16,713 €/QALY (95% CI: − 21,842 to − 12,237) for fibrate in the primary prevention setting. Incremental QALY gains were 0.81 (95% CI: 0.70 to 0.94) for icosapent ethyl and 0.63 (95% CI: 0.52 to 0.74) for fibrate (*p* < 0.001). In the secondary prevention setting, mean ICER were 14,533 €/QALY (95% CI: 9,773 to 19,538) for icosapent ethyl, 115,904 €/QALY (95% CI: 92,004 to 148,286) for evolocumab, and 101,187 €/QALY (95% CI: 86,159 to 118,735) for alirocumab. ICER were negative for the generic drugs. Additional QALY gained were 0.99 (95% CI: 0.83 to 1.14) for icosapent ethyl, 0.87 (95% CI: 0.73 to 1.01) for alirocumab, 0.85 (95% CI: 0.69 to 1.02) for fibrate, and 0.55 (95% CI: 0.42 to 0.69) for evolocumab (all *p* < 0.001).

**Fig. 4:**
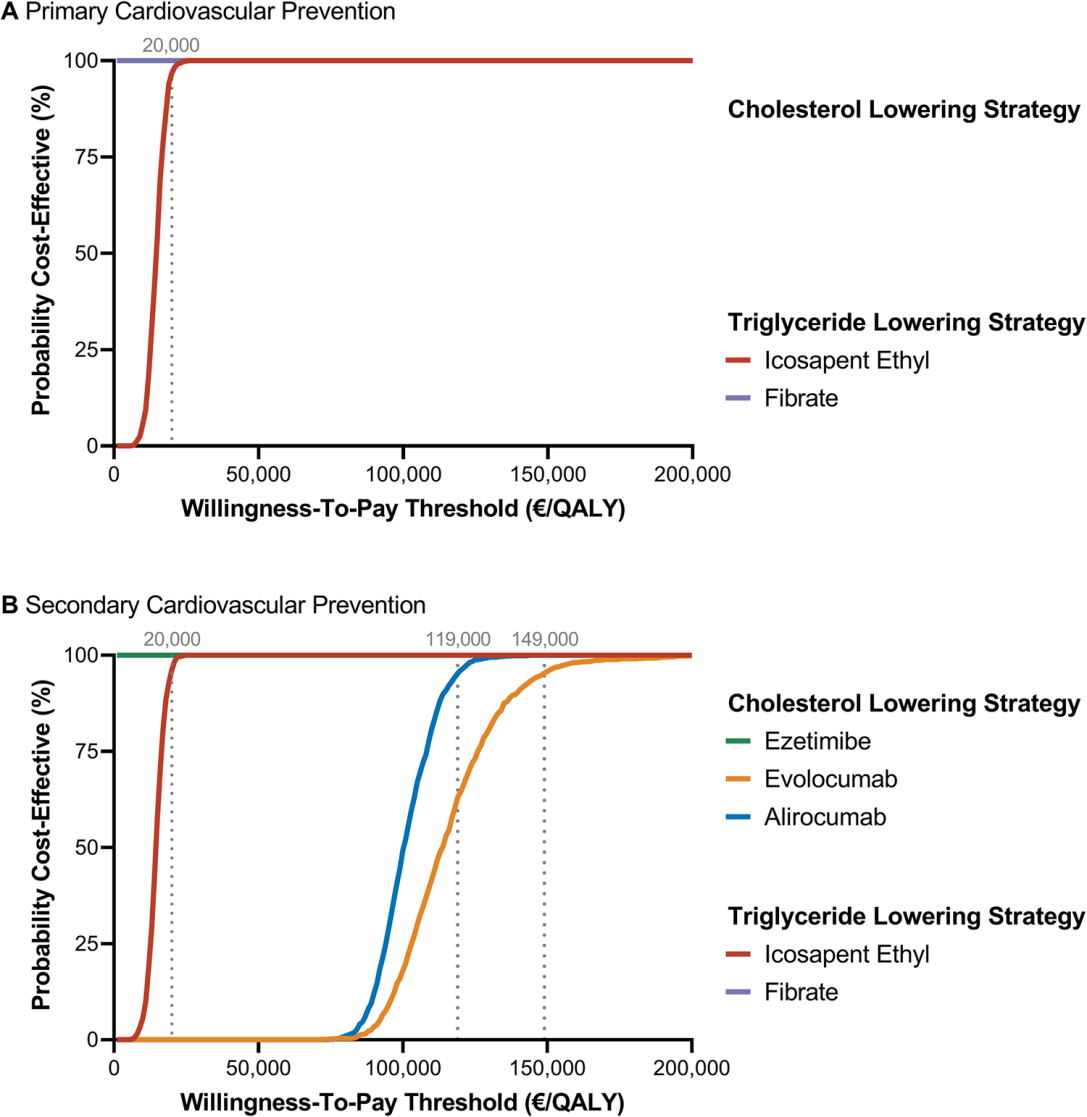
Cost-effectiveness acceptability curves at different willingness-to-pay thresholds for cholesterol and triglyceride lowering treatment strategies in combination with statin. Results are visualized for primary (**A**) and secondary cardiovascular prevention (**B**). All costs are presented in Euros (€) and inflation adjusted to 2021 values. Willingness-to-pay threshold in €/QALY. Dotted lines present the willingness-to-pay threshold at which the probability of cost-effectiveness surpasses 95%. Treatment options were categorized in cholesterol lowering (ezetimibe, alirocumab, evolocumab) and triglyceride lowering (icosapent ethyl, fibrate) strategies based on the European Society of Cardiology guidelines [8]. *QALY* quality-adjusted life year

Results of the WTP analysis are presented in Fig. 4. In the primary prevention cohort, fibrate was cost-effective across all WTP thresholds, whereas icosapent ethyl reached a 95% probability of cost-effectiveness at a WTP threshold of €20,000. In the secondary prevention cohort, the generic drugs ezetimibe and fibrate were cost-effective across all thresholds. A 95% probability of cost-effectiveness was surpassed at €20,000 for icosapent ethyl, €119,000 for alirocumab, and €149,000 for evolocumab.

Scenario and pricing analyses are enclosed in Supplement Table e3 and Figure e4. A 50% discount on list prices reduces evolocumab’s and alirocumab’s ICER to 45,752 and 43,561 €/QALY, respectively. A 50% premium on icosapent ethyl’s list price would yield an ICER of 38,081 and 29,845 €/QALY for primary and secondary prevention. Results varied by discount rate and the annual expected CVD risk increase. Assuming a more aggressive cardiovascular prevention strategy, entailing treatment start at the age of 55 for 25 years, reduces calculated ICER of all drugs. Especially icosapent ethyl’s cost-effectiveness ratio for primary prevention is almost halved from 18,133 to 9,381 €/QALY.

## Discussion and Conclusions

This study evaluated the cost-effectiveness of current lipid-lowering treatment options for primary and secondary cardiovascular prevention from the perspective of the German healthcare system. For primary prevention, results demonstrate that icosapent ethyl plus statin is a cost-effective use of resources (ICER: 18,133 €/QALY). For secondary prevention, ezetimibe (ICER: − 9,555 €/QALY), icosapent ethyl (ICER: 14,485 €/QALY), and the PCSK9 inhibitors evolocumab (ICER: 114,639 €/QALY) and alirocumab (ICER: 100,532 €/QALY) increase patient benefit at different economic costs. Fibrates are viable low-cost alternatives to reduce triglyceride levels.

Before patent expiry, ezetimibe was not assessed cost-effective in the US (ICER: 152,000 USD/QALY) [23]. However, price reductions caused by genericization improved the cost-effectiveness of ezetimibe (ICER: 81,000 USD/QALY) [24]. Studies of ezetimibe conducted in Norway, Finland, Australia, and the UK provide further evidence for the positive benefit-to-expense ratio of ezetimibe in secondary cardiovascular prevention [25–28]. Nevertheless, ezetimibe’s ICER was found to be higher than the WTP thresholds in Thailand and China [29, 30]. Consequently, price reductions caused by generic entry alongside region-specific CVD incidence rates impact the cost-effectiveness of ezetimibe in cardiovascular prevention. In Germany, ezetimibe is available at €156 per year, while both incidence and prevalence of CVD remain high [1, 21]. Consequently, the estimated ICER of € − 9,555 €/QALY for ezetimibe plus statin relative to statin monotherapy is coherent with expectations and previous literature.

Cost-effectiveness evaluations of PCSK9 inhibitors for cardiovascular prevention were previously conducted by scholars and health technology assessment (HTA) agencies around the world. Initially, PCSK9 inhibitors were frequently assessed as cost-ineffective due to high prices in 2015 (US: ICER of 274,000 USD/QALY at annual treatment costs of USD14,350) [31, 32]. However, price discounts to USD5,850 alongside targeted therapy of high-risk patients decreased the ICER to 92,200 USD/QALY by 2020 [23, 33]. Our results confirm that evolocumab and alirocumab are not cost-effective at list prices of €5,880 and €7,509, respectively [21]. Consequently, German statutory insurances negotiated discounts, which may vary in magnitude across regions and insurers. Our analysis suggests that price reductions beyond 50% are necessary for PCSK9 inhibitors to reach the cost-effectiveness provided by icosapent ethyl.

The cost-effectiveness of icosapent ethyl was previously assessed in the USA, Canada, Australia, and Japan [12, 34–38], after several randomized controlled trials demonstrated the dose-dependent benefits of icosapent ethyl in cardiovascular prevention [39, 40]. In the USA, cost-effectiveness estimates range from 18,000 to 36,118 USD/QALY for primary prevention, depending on model structure and simulation assumptions [35, 37]. For secondary prevention, icosapent ethyl was found to be the dominant treatment strategy compared to the standard of care [35]. These results are coherent with our findings that icosapent ethyl is cost-effective in primary prevention, but offers even more value to patients and insurers in secondary prevention. However, cost-effectiveness estimates in Australia and Canada are mixed. Altough the Canadian Agency for Drugs and Technologies in Health (CADTH) demanded a 43% discount based on an ICER of 105,053 CAD/QALY to reach the Canadian WTP of 50,000 CAD/QALY, other studies estimate an ICER of 42,797 CAD/QALY at a treatment cost of CAD3,557 [36, 37]. In Australia, Gao et al. evaluated icosapent ethyl as not cost-effective with an ICER of 59,036 AUD/QALY [38]. In contrast, Ademi et al. assessed icosapent ethyl as cost-effective (ICER: 45,036 AUD/QALY), especially for secondary prevention [12]. Similarly, Koder et al. evaluated eicosapentaenoic acid as cost-effective for secondary, yet not for primary prevention [41]. Nevertheless, their model derives transition probabilities from the JELIS trial which was conducted 20 years ago in Japan [42]. All this shows that the cost-effectiveness of icosapent ethyl is dependent on modelling assumptions alongside drug pricing in regional settings. To the best of our knowledge, this is the first study evaluating the cost-effectiveness of icosapent ethyl in the European context.

Discrepancies in cost-effectiveness ratios for the investigated treatment options are not only driven by differential prices, but also distinct efficacies across cardiovascular event types. While icosapent ethyl, ezetimibe, evolocumab, and alirocumab reduced the risk of MACE by 6 to 25%, the risk reduction was differentially distributed across cardiovascular events with separate costs and disutilities for patients. For instance, the occurrence of non-fatal MI was reduced by 32% (95% CI: 21 to 42) for icosapent ethyl, 28% (95% CI: 19 to 36) for evolocumab, 14% (95% CI: 4 to 23) for alirocumab, and 13% (95% CI: 5 to 20) for ezetimibe. In contrast, the risk reduction for more expensive non-fatal strokes was distributed in a different magnitude and manner: 29% (95% CI: 18 to 40) for icosapent ethyl, 9% (95% CI: − 7 to 22) for evolocumab, 27% (95% CI: 7 to 43) for alirocumab, and 14% (95% CI: 0 to 27) for ezetimibe. These distinct efficacies across cardiovascular event types may ultimately explain why alirocumab provides a greater QALY gain than evolocumab even though their observed overall MACE reduction was the same.

### Limitations

There are some limitations to our analyses. First, we compared treatment options across clinical trials with different inclusion and exclusion criteria, study sites, and time periods. For instance, the ODYSSEY trial includes patients 1 year after hospitalization for acute coronary syndrome with LDL-C level above 70 mg/dL, whereas the IMPROVE-IT trial restricts the time to 10 days with LDL-C levels above 50 mg/dL. However, we derived distinct transition probabilities from each trial to overcome this limitation. Furthermore, concerns were raised about the use of mineral oil as a placebo in the REDUCE-IT trial. Consequently, the efficacy of icosapent ethyl could be upward biased and thus overestimate the cost-effectiveness of icosapent ethyl [5, 43].

Second, transition probabilities were derived from clinical trials with time frames ranging from 2.2 to 6.0 years, subsequently annualized, and then extrapolated to a 20-year simulation with annually increasing risks. Our scenario analysis demonstrates that the annual CVD risk increase impacts ICER outcomes.

Third, cost-effectiveness ratios were evaluated based on the healthcare system of a single country (Germany). Nevertheless, our model’s structure and outcome direction may also inform reimbursement decisions in other European countries, e.g. England, Scotland, Spain, Italy, the Netherlands.

Furthermore, our model neglects side effects of treatment options, such as injection-site reactions for PCSK9 inhibitors or atrial fibrillation, bleeding, and gastrointestinal pain for icosapent ethyl. Additionally, unobserved discounts on list prices negotiated between statutory insurance funds and manufacturers may impact calculated cost-effectiveness ratios as demonstrated in our pricing analysis.

Finally, our model does not analyze the cost-effectiveness of triple or quadruple lipid-lowering treatment options. For instance, patients may receive combination treatments entailing statins, ezetimibe, fibrates, and icosapent ethyl. From a clinical perspective, it is only warranted to compare within cholesterol lowering treatment strategy classes and within triglyceride lowering treatment strategy classes, yet no comparison across classes is advised as they are indicated for different patient groups. Further studies are necessary to assess the impact of therapy sequence on clinical efficacy and costs.

## Supplementary Information

Below is the link to the electronic supplementary material.

Supplementary file1 (PDF 1.25 MB)

## Data Availability

Not applicable.
