# Supplementary material for: Cost-Effectiveness of Lipid-Lowering Therapies for Cardiovascular Prevention in Germany
Source: Cardiovasc Drugs Ther. 2022 Jan 11;37(4):683–94. doi: 10.1007/s10557-021-07310-y (PMC10397126; doi:10.1007/s10557-021-07310-y)
Supplement: Supplementary file 1 — Supplementary file1 (PDF 1.25 MB) [file 10557_2021_7310_MOESM1_ESM.pdf]

Supplementary Online Content

Michaeli DT, Michaeli JC, Boch T, Michaeli T. Cost-Effectiveness of Lipid-Lowering Therapies for Cardiovascular Prevention in Germany. Cardiovascular Drugs and Therapy. 2022. doi:10.1007/s10557-021-07310-y

Table e1 Transition probabilities between health states and for acute cardiovascular events

Table e2 Model input parameters: base case value, variation, and distribution for hazard ratios, utilities, and costs (fibrate)

Table e3 Scenario analysis for the incremental cost-effectiveness ratio (ICER in €/QALY)

Figure e1 Cost-effectiveness plane with efficiency frontier for cholesterol and triglyceride lowering treatment strategies in combination with statin by life years gained

Figure e2 Tornado plots for primary cardiovascular prevention

Figure e3 Tornado plots for secondary cardiovascular prevention

Figure e4 Incremental cost-effectiveness ratios as a function of the annual treatment cost

This supplementary material has been provided by the authors to give readers additional information about their work.

	Icosapent Ethyl		Evolocumab		Alirocumab		Ezetimibe		Fibrate	
	Treatment + Statin	Statin	Treatment + Statin	Statin	Treatment + Statin	Statin	Treatment + Statin	Statin	Treatment + Statin	Statin
**Primary Prevention**										
Non-fatal MI	0.0121	0.0154	NA	NA	NA	NA	NA	NA	0.0094	0.0096
CVD death	0.0064	0.0071	NA	NA	NA	NA	NA	NA	0.0053	0.0058
Non-CVD death	0.0037	0.0032	NA	NA	NA	NA	NA	NA	0.0056	0.0055
Non-fatal stroke	0.0036	0.0045	NA	NA	NA	NA	NA	NA	0.0027	0.0024
Hospitalization for unstable angina	0.0040	0.0052	NA	NA	NA	NA	NA	NA	0.0081	0.0078
Coronary revascularization	0.0142	0.0187	NA	NA	NA	NA	NA	NA	0.0297	0.0282
**Secondary Prevention**										
Non-fatal MI	0.0187	0.0274	0.0148	0.0205	0.0244	0.0283	0.0183	0.0212	0.0215	0.0244
CVD death	0.0099	0.0125	0.0084	0.0080	0.0092	0.0104	0.0102	0.0102	0.0120	0.0146
Non-CVD death	0.0056	0.0056	0.0064	0.0062	0.0036	0.0046	0.0130	0.0132	0.0127	0.0137
Non-fatal stroke	0.0055	0.0078	0.0058	0.0064	0.0042	0.0058	0.0055	0.0065	0.0061	0.0060
Hospitalization for unstable angina	0.0061	0.0091	0.0078	0.0080	0.0014	0.0023	0.0029	0.0027	0.0185	0.0196
Coronary revascularization	0.0220	0.0336	0.0257	0.0330	0.0287	0.0327	0.0385	0.0406	0.0727	0.0775

Table e1 Transition probabilities between health states and for acute cardiovascular events

Transition probabilities were derived from cardiovascular outcome trials for each treatment option: Icosapent ethyl (REDUCE-IT), evolocumab (FOURIER), alirocumab (ODYSSEY), ezetimibe (IMPROVE-IT), and fibrate (ACCORD) [1–5]. *MI* myocardial infarct, *CVD* cardiovascular disease.

Parameter	Fibrate		
	Value	Variation	Ref.
**HAZARD RATIOS**			
**Alive without CVD (primary prevention)**			
Non-fatal MI	0.98	(0.84,1.13)	[5]
CVD death	0.92	(0.78,1.06)	[5]
Non-CVD death	1.03	(0.87,1.18)	[5]
Non-fatal stroke	1.13	(0.96,1.29)	[5]
Hospitalization for unstable angina	1.05	(0.89,1.21)	[5]
Coronary revascularization	1.06	(0.90,1.21)	[5]
**Alive with CVD (secondary prevention)**			
Non-fatal MI	0.88	(0.75,1.01)	[5]
CVD death	0.82	(0.70,0.95)	[5]
Non-CVD death	0.92	(0.79,1.06)	[5]
Non-fatal stroke	1.01	(0.86,1.17)	[5]
Hospitalization for unstable angina	0.94	(0.80,1.08)	[5]
Coronary revascularization	0.94	(0.80,1.08)	[5]
**UTILITIES**			
**Alive without CVD**			
65-70 years	0.65	(0.64,0.67)	[6]
70+ years	0.63	(0.62,0.65)	[6]
**Alive with CVD**			
65-70 years	0.57	(0.56,0.59)	[7]
70+ years	0.55	(0.54,0.57)	[7]
**Decrements**			
Non-fatal MI	0.04	(0.02,0.05)	[8]
Non-fatal stroke	0.12	(0.09,0.16)	[8]
Hospitalization for unstable angina	0.09	(0.06,0.13)	[8]
Coronary revascularization	0.01	(0.01,0.03)	[8]
**COSTS**			
Alive without CVD	4,522	(±25%)	[9]
Alive with CVD	8,008	(±25%)	[10,11]
Non-fatal MI	8,588	(±25%)	[11]
CVD death	11,842	(±25%)	[10–13]
Non-CVD death	3,924	(±25%)	[12–14]
Non-fatal stroke	10,441	(±25%)	[11]
Hospitalization for unstable angina	4,791	(±25%)	[10]
Coronary revascularization	9,901	(±25%)	[10]
Annual treatment cost	130		[15]
Annual statin cost	132		[15]
**OTHERS**			
Discount rate	0.03	(0.00,0.10)	[16]
Annual CVD risk increase	0.14	(±25%)	[17]
Annual non-CVD risk increase	0.10	(±25%)	[17]

Table e2 Model input parameters: base case value, variation, and distribution for hazard ratios, utilities, and costs (fibrate)

The table displays each drug's key input parameters used for the base case scenario of the cost-effectiveness model. Variation columns display upper and lower bound of parameter values that were used for sensitivity analysis. For hazard ratios and utilities, this variation equates to 95% confidence interval extracted from cited references. All costs are presented in Euros (€) and are inflation adjusted to 2021 values. Transition probabilities for the model are presented in Supplement Table e1. *CVD* cardiovascular disease, *MI* myocardial infarct, *NA* not applicable.

Scenario	Primary Prevention		Secondary Prevention				
	Icosapent Ethyl	Fibrate	Icosapent Ethyl	Evolocumab	Alirocumab	Ezetimibe	Fibrate
Annual Treatment Cost							
Base Case	18,133	-16,632	14,485	114,639	100,532	-5,584	-10,305
-50%	-1,815	-18,000	-875	45,752	43,561	-9,059	-11,171
+50%	38,081	-15,264	29,845	183,525	157,503	-2,108	-9,440
Discount rate							
3% (Base Case)	18,133	-16,632	14,485	114,639	100,532	-5,584	-10,305
0%	10,973	-16,986	9,642	87,203	80,034	-6,456	-10,208
10%	45,014	-14,925	31,472	219,622	172,130	-1,940	-10,234
Yearly CVD risk increase							
+14% (Base Case)	18,133	-16,632	14,485	114,639	100,532	-5,584	-10,305
+10.5%	43,087	-9,516	27,470	200,370	149,672	1,796	-5,955
+17.5%	5,211	-20,269	6,405	67,249	68,229	-10,261	-12,935
Yearly non-CVD risk increase							
+10% (Base Case)	18,133	-16,632	14,485	114,639	100,532	-5,584	-10,305
+7.5%	18,001	-19,646	14,099	121,163	103,476	-8,810	-12,878
+12.5%	18,215	-13,149	14,947	106,353	96,752	-2,187	-7,319
Early lipid lowering treatment							
Start at 55 for 25 years	9,381	-13,070	9,822	71,899	70,379	-6,348	-7,194

Table e3 Scenario analysis for the incremental cost-effectiveness ratio (ICER in €/QALY)

All costs are presented in Euros (€) and were inflation adjusted to 2021 values. *CVD* cardiovascular disease, *ICER* incremental cost-effectiveness ratio, *QALY* quality-adjusted life year.

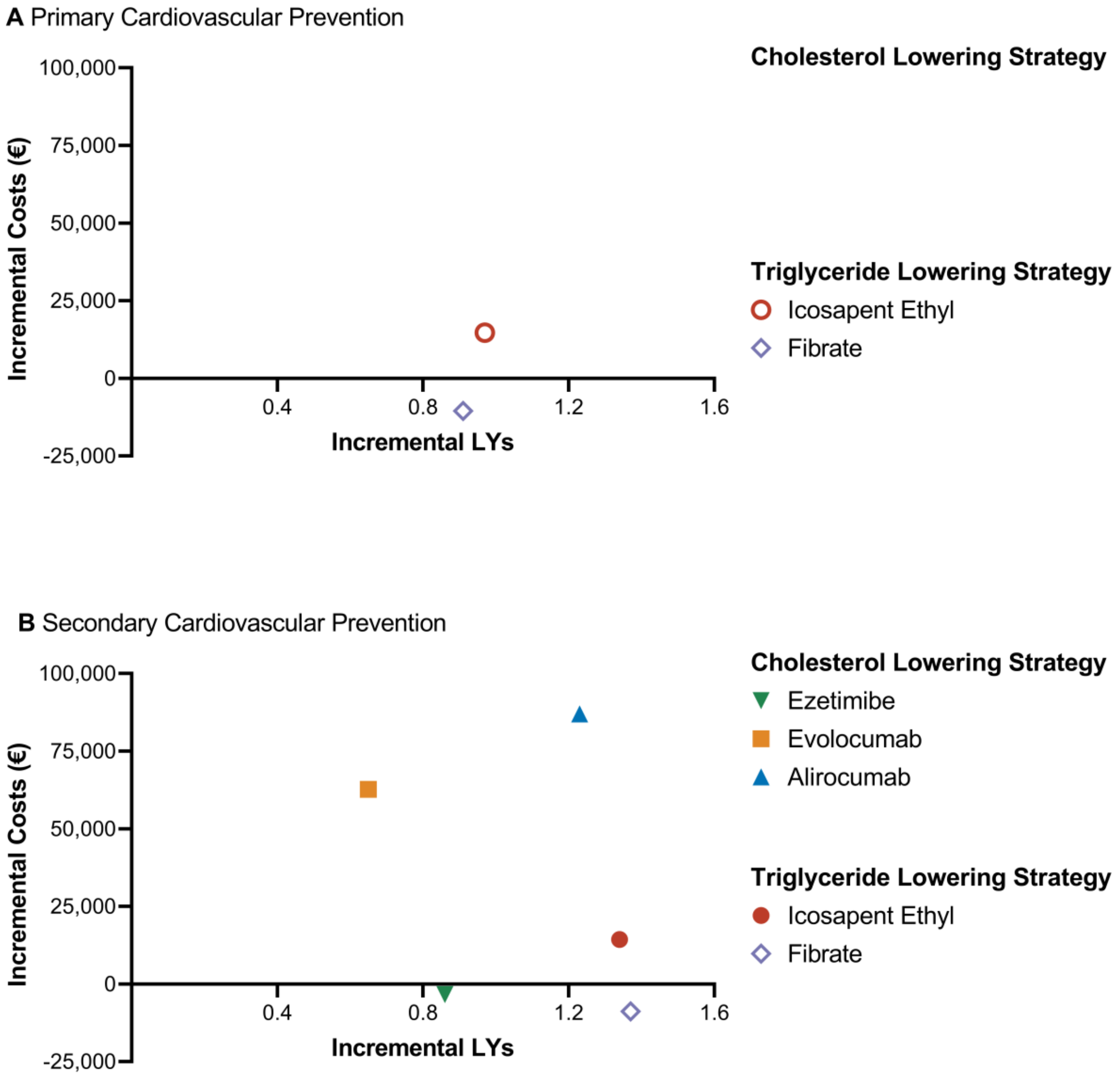

Figure e1 Cost-effectiveness plane with efficiency frontier for cholesterol and triglyceride lowering treatment strategies in combination with statin by life years gained

Resulats are visualized for primary (**A**) and secondary cardiovascular prevention (**B**). All costs are presented in Euros (€) and were inflation adjusted to 2021 values. LYs and costs are displayed per person. Treatment options were categorized in cholesterol lowering (ezetimibe, alirocumab, evolocumab) and triglyceride lowering (icosapent ethyl, fibrate) strategies based on the European Society of Cardiology guidelines [18]. *LY* life year.

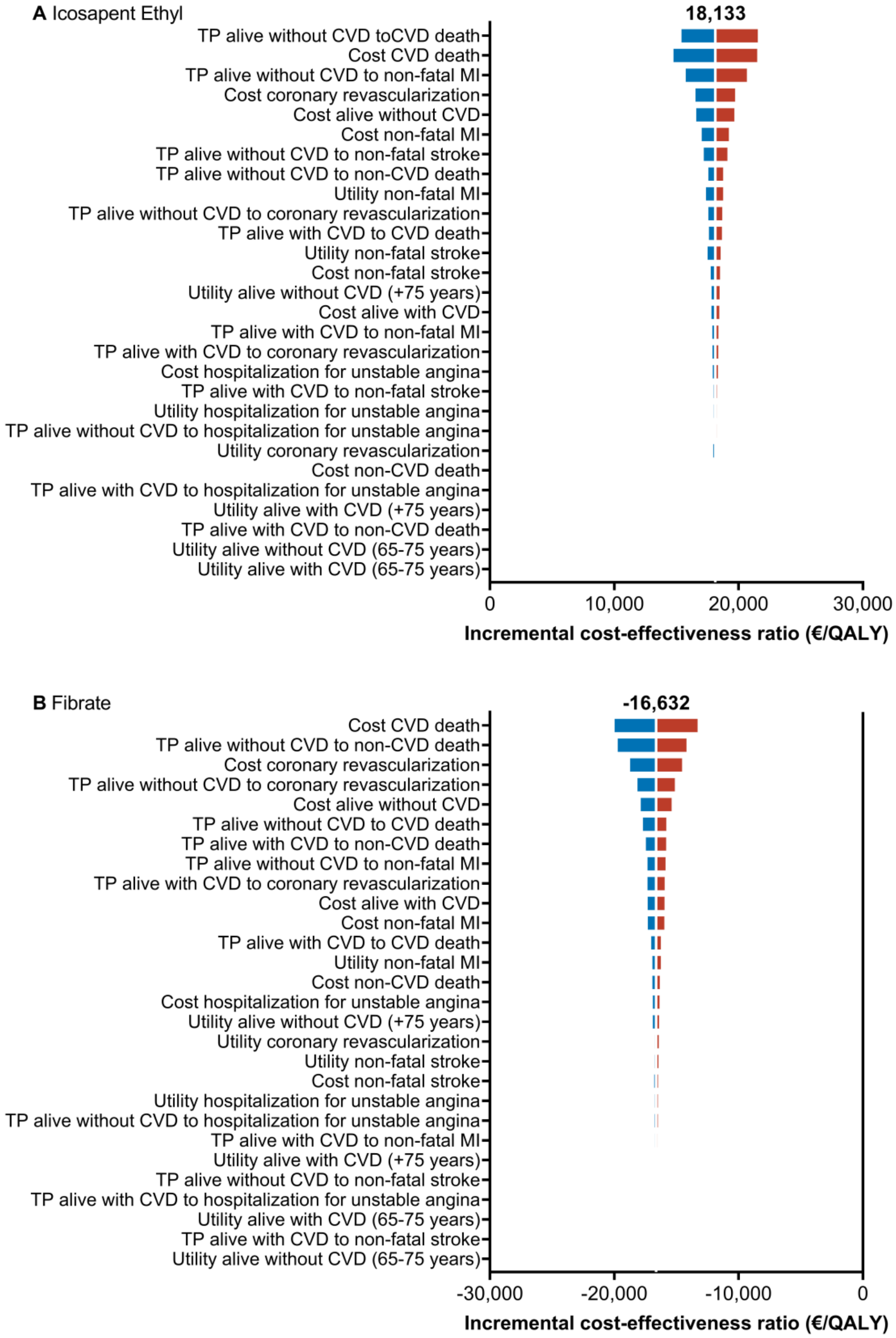

Figure e2 Tornado plots for primary cardiovascular prevention

Results are visualized for icosapent ethyl (**A**) and fibrate (**B**). Input parameters were varied by 95% confidence intervals displayed in Table 1. All costs are presented in Euros (€) and were inflation adjusted to 2021 values. *CVD* cardiovascular disease, *MI* myocardial infarct, *TP* transition probability.

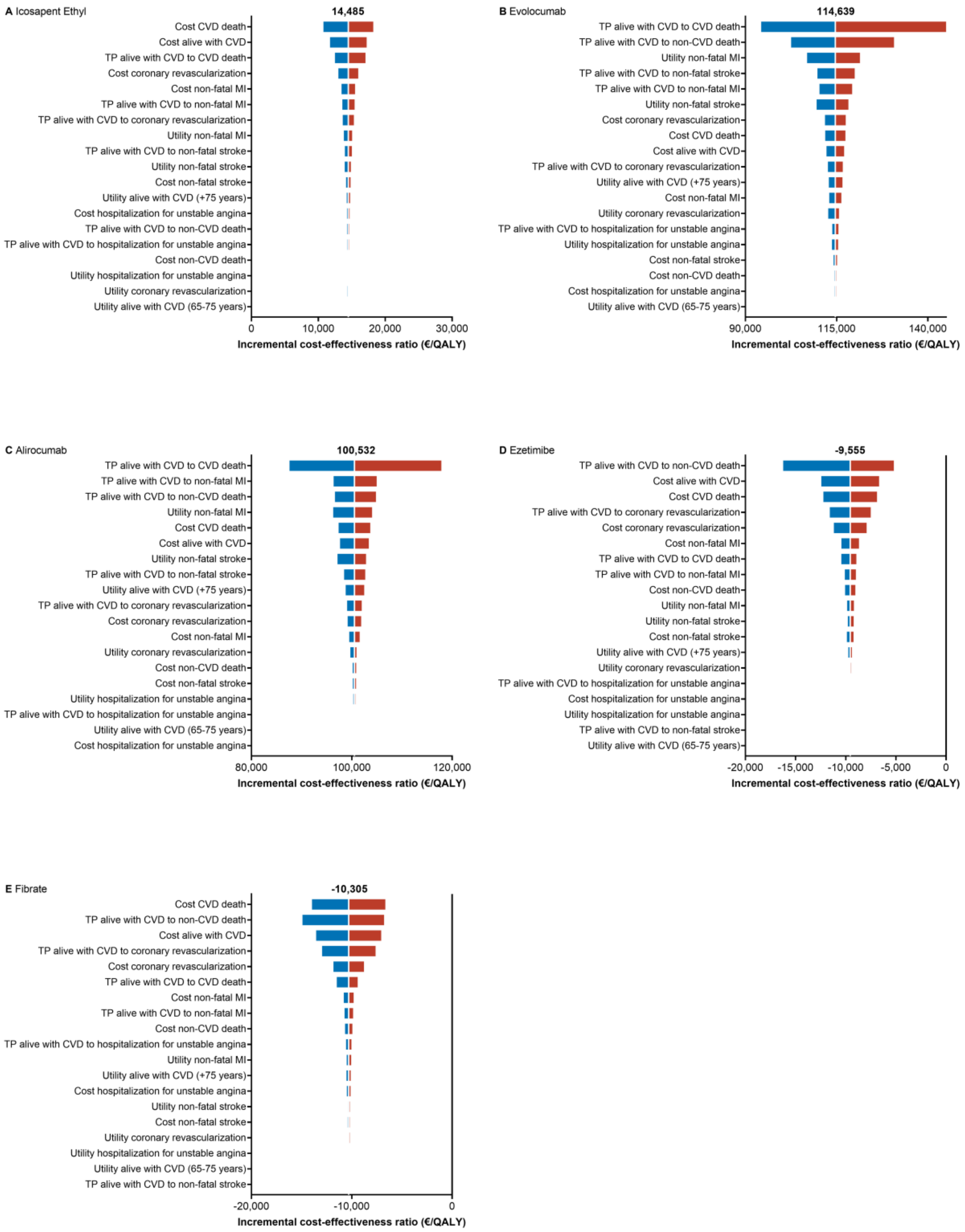

Figure e3 Tornado plots for secondary cardiovascular prevention

Results are displayed for icosapent ethyl (**A**), evolocumab (**B**), alirocumab (**C**), ezetimibe (**D**), and fibrate (**E**). Input parameters were varied by 95% confidence intervals displayed in Table 1. All costs are presented in Euros (€) and were inflation adjusted to 2021 values. *CVD* cardiovascular disease, *MI* myocardial infarct, *TP* transition probability.

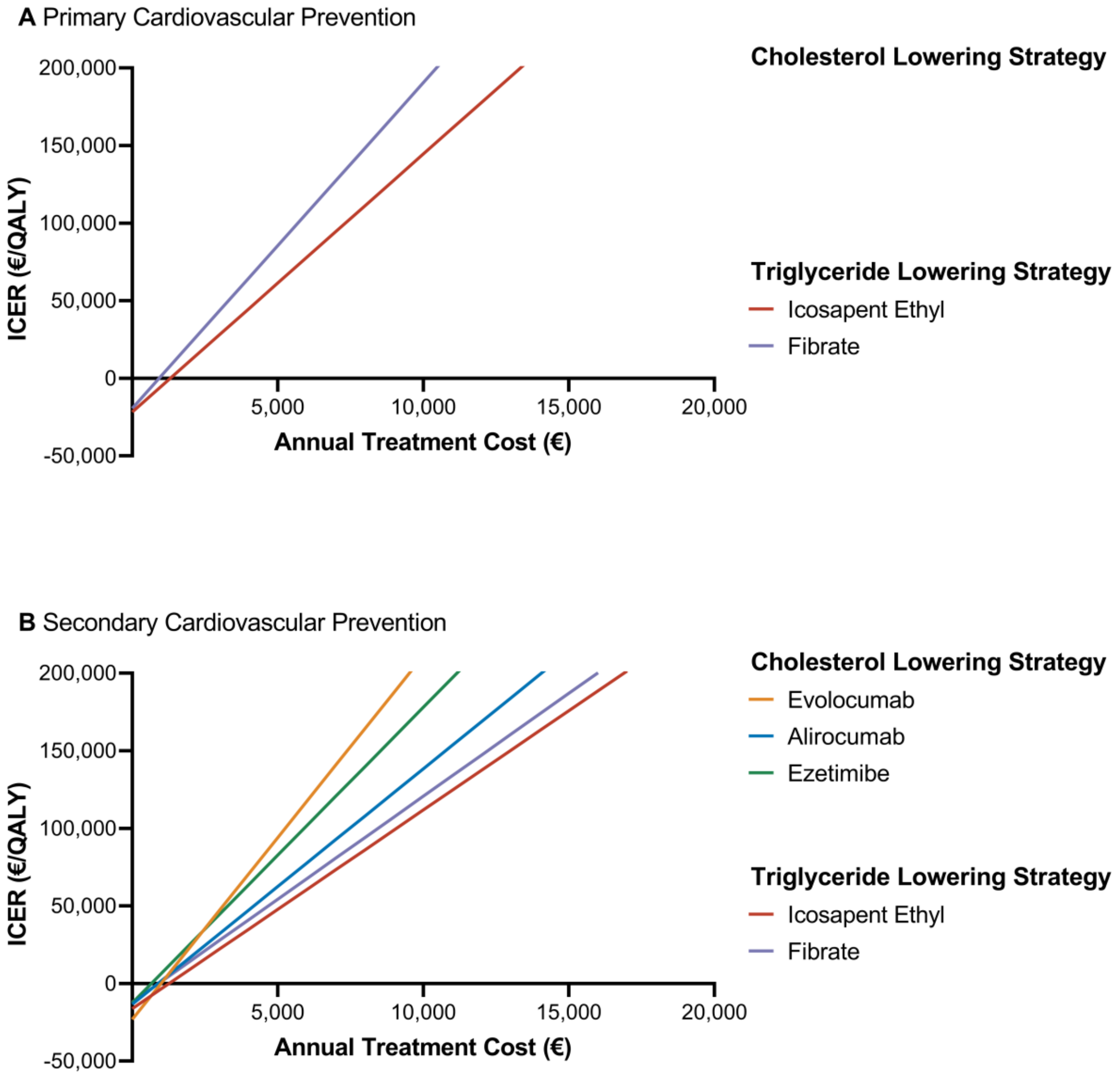

Figure e4 Incremental cost-effectiveness ratios as a function of the annual treatment cost

Results are visualized for primary (**A**) and secondary cardiovascular prevention (**B**). All costs are presented in Euros (€) and were inflation adjusted to 2021 values. Costs and ICER are displayed per person. Treatment options were categorized in cholesterol lowering (ezetimibe, alirocumab, evolocumab) and triglyceride lowering (icosapent ethyl, fibrate) strategies based on the European Society of Cardiology guidelines [18]. *ICER* incremental cost-effectiveness ratio, *QALY* quality-adjusted life year.

References

1. Bhatt DL, Steg PG, Miller M *et al.* Cardiovascular Risk Reduction with Icosapent Ethyl for Hypertriglyceridemia. *N Engl J Med* 2019;**380**:11–22.

2. Sabatine MS, Giugliano RP, Keech AC *et al.* Evolocumab and Clinical Outcomes in Patients with Cardiovascular Disease. *N Engl J Med* 2017;**376**:1713–22.

3. Schwartz GG, Steg PG, Szarek M *et al.* Alirocumab and Cardiovascular Outcomes after Acute Coronary Syndrome. *N Engl J Med* 2018;**379**:2097–107.

4. Cannon CP, Blazing MA, Giugliano RP *et al.* Ezetimibe Added to Statin Therapy after Acute Coronary Syndromes. *N Engl J Med* 2015;**372**:2387–97.

5. ACCORD Study Group, Ginsberg HN, Elam MB *et al.* Effects of combination lipid therapy in type 2 diabetes mellitus. *N Engl J Med* 2010;**362**:1563–74.

6. Ellert U, Kurth BM. [Health related quality of life in adults in Germany: results of the German Health Interview and Examination Survey for Adults (DEGS1)]. *Bundesgesundheitsblatt* 2013;**56**:643–9.

7. Lewis EF, Li Y, Pfeffer MA *et al.* Impact of Cardiovascular Events on Change in Quality of Life and Utilities in Patients After Myocardial Infarction: A VALIANT Study (Valsartan In Acute Myocardial Infarction). *JACC Heart Fail* 2014;**2**:159–65.

8. Kazi DS, Moran AE, Coxson PG *et al.* Cost-effectiveness of PCSK9 Inhibitor Therapy in Patients With Heterozygous Familial Hypercholesterolemia or Atherosclerotic Cardiovascular Disease. *JAMA* 2016;**316**:743–53.

9. Wille E, Scholze J, Alegria E *et al.* Modelling the costs of care of hypertension in patients with metabolic syndrome and its consequences, in Germany, Spain and Italy. *Eur J Health Econ HEPAC Health Econ Prev Care* 2011;**12**:205–18.

10. Schmid T. Costs of treating cardiovascular events in Germany: a systematic literature review. *Health Econ Rev* 2015;**5**, DOI: 10.1186/s13561-015-0063-5.

11. Kähm K, Laxy M, Schneider U *et al.* Health Care Costs Associated With Incident Complications in Patients With Type 2 Diabetes in Germany. *Diabetes Care* 2018;**41**:971–8.

12. Marie Curie Cancer Care. Understanding the cost of end of life care in different settings. 2013.

13. Sauer S, Müller R, Rothgang H. [Institutionalised dying in Germany. Trends in place of death distribution: home, hospitals and nursing homes]. *Z Gerontol Geriatr* 2015;**48**:169–75.

14. Winnat C. [Costs of a hospital stay]. *Ärzte Ztg* 2018.

15. AOK Niedersachsen. [Price list lipid-lowering agents]. 2021.

16. IQWiG. *[General Methods - Version 5.0]*. Cologne: Institute for Quality and Efficiency in Health Care, 2017.

17. Destatis. [Mortality table (period mortality table): Germany, years, sex, full age, Federal Statistical Office]. 2021.

18. Mach F, Baigent C, Catapano AL *et al.* 2019 ESC/EAS Guidelines for the management of dyslipidaemias: lipid modification to reduce cardiovascular risk: The Task Force for the management of dyslipidaemias of the European Society of Cardiology (ESC) and European Atherosclerosis Society (EAS). *Eur Heart J* 2020;**41**:111–88.
